# *In Situ* Hybrid Solid-State Electrolytes for Lithium Battery Applications

**DOI:** 10.1021/acsapm.4c00473

**Published:** 2024-04-26

**Authors:** Natalia Stankiewicz, Miryam Criado-Gonzalez, Jorge L. Olmedo-Martínez, Eider Matxinandiarena, Pedro López-Aranguren, Francisco Bonilla, Grazia Accardo, Damien Saurel, Didier Devaux, Irune Villaluenga

**Affiliations:** † POLYMAT, Applied Chemistry Department, Faculty of Chemistry, University of the Basque Country (UPV/EHU), Paseo Manuel de Lardizábal 3, 20018 Donostia-San Sebastián, Spain; ‡ Basque Research and Technology Alliance, Parque Tecnológico de Alava, Center for Cooperative Research on Alternative Energies (CIC EnergiGUNE), Albert Einstein 48, 01510 Vitoria-Gasteiz, Spain; § Laboratoire d’Electrochimie et de Physicochimie des Matériaux et des Interfaces, Université Grenoble Alpes, Université Savoie Mont Blanc, CNRS, Grenoble INP, F-38000 Grenoble, France; ∥ Ikerbasque, Basque Foundation for Science, Plaza Euskadi 5, 48009 Bilbao, Spain

**Keywords:** all-solid-state battery, electrolytes, inorganics, polymers, hybrids

## Abstract

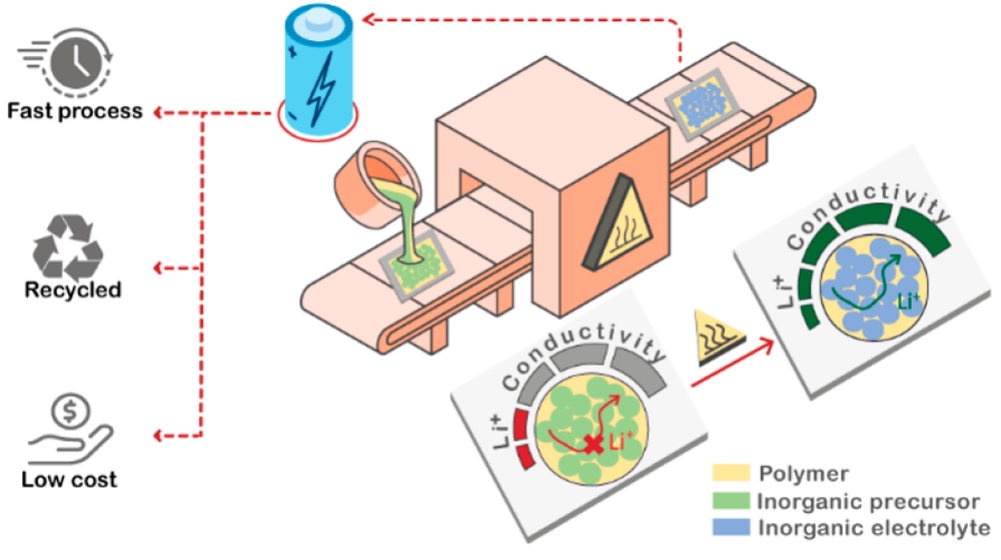

The translation of inorganic–polymer hybrid battery materials from laboratory-scale to industry-relevant battery manufacturing processes is difficult due to their complexity, scalability, and cost and the limited fundamental knowledge that is available. Herein, we introduce a unique and compelling approach for the preparation of hybrid solid electrolytes based on an *in situ* synthesized halide electrolyte (Li_3_InCl_6_) in the presence of a non-conducting polymer (styrene–ethylene–butylene–styrene block copolymer). This innovative *in situ* approach delivers flexible self-standing membranes with good ionic conductivity (0.7 × 10^–4^ S/cm at 30 °C) and low activation energy (0.25 eV). This study suggests that the total conductivity is dominated by the inorganic–polymer interfaces and the microstructure of the hybrids affects the energy barriers to ion transport. This work opens a promising sustainable and cost-efficient route that can be easily implemented in current battery manufacturing lines.

## 1 Introduction

All solid-state batteries (ASSBs) have been identified as a game-changing technology for developing high-performance energy storage systems that are safer and more sustainable to reach the goals of a carbon-neutral society.^1,2^ Solid-state electrolytes (SSEs) are essential for their development because they may replace the flammable liquid electrolytes that pose serious safety concerns in currently available batteries.^3−5^ Their practical application enable higher energy density and improved cyclability.^6^

Nowadays, SSEs based on inorganic materials, mostly oxides, sulfides, and halides, have been deeply investigated. The reported ionic conductivity values for these electrolytes exceed 1 mS/cm at room temperature (RT). Oxide-based electrolytes, like Li_3*x*_La_2/3*x*_TiO_3_, Li_7_La_3_Zr_2_O_12_, or (Li_1+*x*_Al_*x*_Ge_2–*x*_(PO_4_)_3_, show good mechanical properties, i.e., high moduli and wide electrochemical windows.^7,8^ However, they require elevated sintering temperatures, which limits their practical application. Besides, they are challenging to process and prone to breakage due to their brittleness.^9,10^ Sulfide-based SSEs, for example, Li_10_GeP_2_S_12_, glass-ceramic Li_2_S–P_2_S_5_, and Li_6_PS_5_Cl, also present excellent ionic transport properties, although one of their main drawbacks is their high reactivity when in contact with water, in which harmful hydrogen sulfide is released, which hampers their upscalability.^11−13^ However, among SSEs based on various anion chemistry like oxides or sulfides, halide-based electrolytes present larger ionic radii of the anions, leading to higher ion mobility and excellent deformability.^14^ Monovalent halogen anions have weaker Coulombic interactions with lithium ions compared to divalent sulfur or oxygen anions during ion migration, which contributes to the faster kinetics of lithium ions and higher ionic conductivity. Moreover, halide anions possess a greater electrochemical redox potential than oxide and sulfide anions, resulting in improved oxidative stability.^15^ In particular, a lithium indium chloride (LIC) electrolyte has attracted considerable attention because it is composed of non-critical raw materials, presents high ionic conductivity values (reportedly up to 2 mS/cm at RT), and can be obtained by an environmentally friendly and cost-effective water-based synthesis. A LIC electrolyte can be synthesized by simple solvent evaporation at temperatures significantly lower than the temperatures required by conventional sintering processes, and it is electrochemically stable at high voltages.^16−18^ However, halide-based electrolytes are known to be unstable in ambient air. Although LIC forms a stable hydrate having low conductivity, its high Li^+^ conductivity can be easily recovered by a heating step, which is propitious in terms of recyclability possibilities.^19^ This solution may decrease manufacturing costs and find practical application, which is promising for the design of battery production lines.

The combination of inorganic electrolytes and polymers in hybrid solid electrolytes (HSEs) with inorganic-rich content has growing interest in an attempt to achieve compatible production technology capable of scaling up and fulfilling the requirements of the automotive market.^20^ HSEs combine the advantages of inorganic electrolytes (high ionic conductivity at RT, single-ion properties, etc.) and polymer binders (flexibility, malleability, etc.). This approach of combining materials to favor their advantageous properties and overcome their limitations is also appealing with the current trends of designing battery components for electric vehicle application.^21^ The main strategy of HSEs is to disperse an inorganic electrolyte into a polymer matrix.^20,22−25^ For this, the inorganic electrolyte is previously synthesized, which adds numerous steps for the synthesis/process and, therefore, the cost of the hybrid electrolytes. There is a demand by battery manufacturers for the development of more sustainable, energy- and cost-efficient synthetic techniques and processes. To that aim, cost-effective one-pot synthesis meets the intrinsic advantages of sustainability, simple operation, high mass efficiency, and less waste disposal.^26,27^ The design of a battery component is a multidisciplinary effort to meet market demand and develop next-generation batteries.^28^ In this study, we introduce an innovative one-pot approach for the preparation of HSEs based on an *in situ* synthesized inorganic electrolyte (Li_3_InCl_6_) in the presence of a polymer matrix (styrene–ethylene–butylene–styrene block copolymer). We compare this approach to conventional physical mixture electrolyte preparation (blends). The *in situ* inorganic–polymer hybrid electrolytes present the same range of ionic conductivity and thermal and mechanical properties as the blend electrolytes, validating the efficiency of our innovative approach, which permits one to reduce the number of fabrication steps, time, and energy for a cost-efficient battery manufacturing.

## 2 Experimental Section

### 2.1 Chemicals

Lithium chloride (LiCl; 99.9%, ultra dry) and indium(III) chloride (InCl_3_; 99.999%, anhydrous) were obtained from Alfa Aesar. Styrene–ethylene–butylene–styrene (SEBS; *M*_w_ = 18000 g/mol) with a 29:71 mole ratio of styrene–rubber was obtained from Sigma-Aldrich. Anhydrous toluene was obtained from Scharlab. All of the reagents were used without further purification and under a glovebox atmosphere.

### 2.2 Material Synthesis

#### 2.2.1 Inorganic Precursor and LIC Synthesis

The inorganic phase was synthesized by the sol–gel method. LiCl and InCl_3_ were weighed in a stoichiometric ratio inside the glovebox. In an ambient environment, the reagents were dissolved in deionized water. The solution was dried with a rotavapor, subsequently fully dried under high vacuum at 40 °C overnight to obtain the inorganic precursor (HLIC), and transferred to the glovebox. LIC was synthesized by drying the inorganic precursor at 200 °C for 5 h under high vacuum. HLIC and LIC were ground in a mortar before further use.

#### 2.2.2 Hybrid Electrolytes Preparation

Motivated by the recent research on the polymer interactions with the inorganic particles in HSEs compositions, a non-conducting SEBS copolymer was chosen as the organic component of the electrolyte.^22,29,30^ The thermoplastic elastomer with excellent mechanical properties (thanks to the hard polystyrene part and rubbery ethylene–butylene domains) for self-standing membrane preparation is nonreactive, preserving any side reactions with the inorganic material. Moreover, SEBS presents high thermal stability, which is necessary for the *in situ* synthesis of the Li_3_InCl_6_ electrolyte that is carried out at 200 °C. Furthermore, one of the criteria for the slurry-based processing of the SSE is the choice of a good solvent. Toluene was selected as the solvent due to its compatibility with the SEBS polymer and its nonreactivity with the inorganic electrolyte. The *in situ* HSE was prepared by weighing HLIC (recalculated for LIC wt % in the final composition) and dispersing it in toluene with 1 g/mL of inorganic precursor concentration inside the glovebox. After stirring for 2 h at RT, a 15 wt % SEBS solution was added to the HLIC dispersion and stirred overnight. After the mixture was poured into a Teflon Petri dish and predried at 40 °C on a hot plate, the membrane was further dried at 200 °C for 5 h under high vacuum. The preparation of a blend HSE was done by mixing the LIC dispersion in toluene (1 g/mL) with a SEBS solution, casting into a Teflon Petri dish, and drying overnight at RT under vacuum. Finally, the last step was hot-pressing of the dried membranes (*in situ* and blend HSEs) at 200 °C for 30 min at 150 MPa.

### 2.3 Material Characterization

X-ray diffraction (XRD; Bruker D8 Discover X-ray diffractometer, Cu Kα_1_, λ = 1.54056 Å, radiation in the 2θ range 10–80° with a step width of 0.02044°) was used to examine the crystallinity of the inorganic material. The structure of the polymer was checked by ^1^H NMR (Avance III 400 MHz digital NMR spectrometer). Samples were placed in a NMR tube inside the glovebox, and CDCl_3_ solvent was later added in ambient air. The morphology of the electrolytes was investigated by high-resolution field-emission-gun scanning electron microscopy (SEM), applying a 10 kV acceleration voltage. Samples were prepared in a glovebox and then transferred to a scanning electron microscope in a transfer holder without exposure to ambient air. The thermal properties were studied by differential scanning calorimetry (DSC; PerkinElmer DSC 8000) in a range from −40 to +240 °C with a 10 °C/min heating rate under a N_2_ atmosphere and thermogravimetric analysis (TGA; PerkinElmer TGA 8000) from 40 to 800 °C under a N_2_ atmosphere at 10 °C/min. The samples for DSC were placed in aluminum pans inside the glovebox using approximately 5 mg of sample. The rheological properties of the membranes were determined using an ARES-G2 rheometer (TA Instruments) at 40 °C and under a N_2_ atmosphere. Frequency sweeps were performed from 0.1 to 600 rad/s at a fixed oscillation strain of 1%, and oscillatory stress sweeps were carried out from 800 to 14000 Pa at a fixed angular frequency of 6.3 rad. The flammability was assessed using pyrolysis combustion flow calorimetry (PCFC) provided by Fire Testing Technology. For this purpose, the samples were heated at a rate of 1 °C/s until they reached a temperature of 700 °C. A controlled flow of oxygen gas, at a rate of 100 mL/min and comprising 20 vol % in nitrogen, was introduced to the combustion chamber maintained at 900 °C. Small-angle X-ray scattering (SAXS) was performed at beamline BL11-NCD, ALBA Synchrotron, Cerdanyola del Vallés, Barcelona, Spain.

### 2.4 Electrochemical Characterization

The total ionic conductivity of the membranes was measured by electrochemical impedance spectroscopy (EIS). The frequency range was 7 MHz to 1 Hz with a signal amplitude of 40 mV. The EIS spectra were collected from 30 to 90 °C in 10 °C increments, and the ionic conductivity (σ) was calculated from the equation
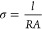
where *l* is the membrane thickness (cm), *A* is the active surface area (cm^2^), and *R* is the total resistance (Ω).

The activation energy of the electrolytes was calculated using the equation
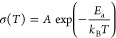
where *A* is the preexponential constant, *E*_a_ is the energy of activation calculated from the slope of the Arrhenius plot (eV), *k*_B_ is the Boltzmann constant (J/K), and *T* is the temperature (K).

## 3 Results and Discussion

**Figure 1:**
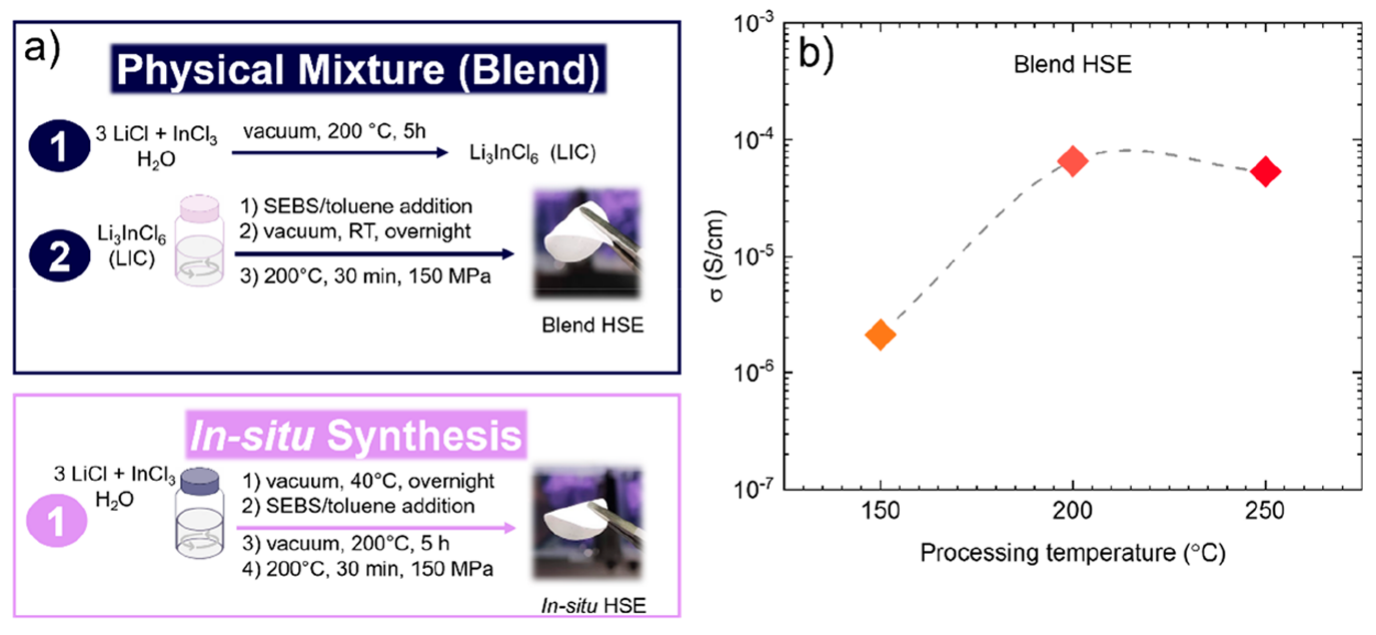
(a) Schematic presentation of two approaches [*in situ* synthesis and conventional physical mixture (blend)] for HSEs preparation and (b) ionic conductivity of the blend HSE as a function of the processing temperature.

We focus on two samples, which are named *in situ* HSE and blend HSE (Figure 1a). Following the work of Sun *et al.* for the synthesis of the Li_3_InCl_6_ electrolyte,^16^ a traditional physical mixture (blend HSE) of a HSE composed of 80 wt % Li_3_InCl_6_ and 20 wt % SEBS was prepared. The polymer amount (SEBS) was chosen to be 20 wt % (42 vol %) in order to obtain a HSE with flexible properties at low thickness (<50 μm). For this, the inorganic compound, Li_3_InCl_6_, was synthesized independently, dispersed in toluene, and mixed with a solution of SEBS in toluene (15 wt %). Once the mixture was casted and solvent was evaporated, the resulting HSE membrane was obtained (23 μm). This conventional method was then compared to the innovative *in situ* approach. The *in situ* HSE synthesis technique involved a mixture of Li_3_InCl_6_ precursors (LiCl and InCl_3_ were mixed to have a homogeneous mixture) with a SEBS block copolymer solution in toluene. The resulting mixture was then casted and dried in order to get a flexible and thin membrane (48 μm), which was subsequently heated at 200 °C for 5 h to obtain the ion-conducting crystalline phase of Li_3_InCl_6_ in the presence of the SEBS block copolymer. The amount of inorganic precursor that was used for the *in situ* approach was calculated for the final 80 wt % (58 vol %) of pure Li_3_InCl_6_ in the composition.

After the HSE membranes were casted and dried, a hot press step was performed. This step is crucial to achieve intimate contact between the inorganic particles and, thus, the optimized ion-transport properties of HSEs. The temperature effect of the hot press on the ionic conductivity of blend HSE was studied in order to determine the processing temperature, which will be used for both blend and *in situ* HSEs. Three different temperatures [150, 200, and 250 °C, higher than the *T*_g_ values (>120 °C) of the polymers (polystyrene, polyethylene, and polybutylene) that compose the SEBS block copolymer] were investigated, as shown in Figure 1b. For each temperature, the membrane was hot-pressed for 30 min under the same pressure (150 MPa), and after cooling, the ionic conductivities were measured at RT. When blend HSE was heated at 150, 200, and 250 °C under 150 MPa, the ionic conductivities at RT were 2.11 × 10^–6^, 6.58 × 10^–5^ and 5.34 × 10^–5^ S/cm, respectively. The increase of the processing temperature from 150 to 200 °C led to an increase by a factor of about 30 of the ionic conductivity, and then it slightly decreased when the HSE was processed at 250 °C. We attribute this difference in the ionic conductivities to the better contact between the inorganic particles when the SEBS block copolymer is squeezed under temperature and pressure, enabling faster lithium hopping.^31^ The optimized processing temperature was 200 °C, which led to conductivities close to 10^–4^ S/cm at RT. For that reason, the temperature of 200 °C was chosen for both blend HSE and *in situ* HSE processing (Figure 1b). This processing step decreased the thickness of *in situ* HSE by 55% and that of blend HSE by 20%, leading to materials with similar final thicknesses. We attribute the greater thickness reduction of *in situ* HSE to the larger volume of HLIC used for the synthesis and subsequent water evaporation during the LIC *in situ* synthesis. That leads to the more porous membrane from the *in situ* synthesis, which is later able to be compressed more than blend HSE. It is important to highlight that the ability to process thin solid electrolytes on a large scale is critical for developing low-cost solid-state batteries that can deliver practical energy densities. As a consequence, a solvent-casting method was used for electrolytes preparation, which can be scalable in roll-to-roll coaters by the current battery manufacturers.^23^

**Figure 2:**
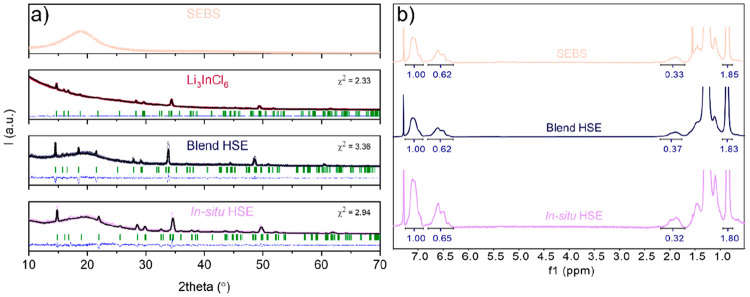
(a) XRD patterns for SEBS, pure LIC, blend HSE, and *in situ* HSE. The collected data are shown as colored circles, the results of the refinement are given as black solid lines, the vertical green bars show the Bragg peaks’ positions for the refined phase *C*12/*m*1, and the differences between the data and refinement results are shown as blue solid lines. Inset: χ^2^ values for the refinements. (b) ^1^H NMR spectra for pure SEBS, blend HSE, and *in situ* HSE.

The crystallographic and chemical compositions of blend and *in situ* HSEs were studied by XRD and NMR techniques. The XRD patterns of blend and *in situ* HSEs match those of the pure crystalline Li_3_InCl_6_ structure with corresponding characteristic peaks at 14, 28, 29, 34, and 49°, confirming the successful synthesis of the inorganic material in the hybrid electrolyte (Figure 2a). The Rietveld refinement results indicate that Li_3_InCl_6_ has a monoclinic symmetry with the *C*2/*m* space group.^16,17^ Low χ^2^ values (2.33, 3.36, and 2.94 for pure LIC, blend HSE, and *in situ* HSE, respectively) indicate the accuracy of the fittings and confirm of intact LIC structure in HSEs. SEBS does not present a crystalline structure; therefore, it does not influence the spectra of the HSEs. ^1^H NMR measurements were performed to ensure that the *in situ* approach did not degrade the SEBS block copolymer. ^1^H NMR spectra for blend and *in situ* HSEs show peaks at 0.85 and 1.88 ppm, which correspond to the aliphatic ethylene/butylene block, and signals at 6.55 and 7.11 ppm, which are associated with aromatic protons from the polystyrene chain (Figure 2b). ^1^H NMR and XRD characterizations verify that the *in situ* synthesis of the halide electrolyte in the presence of a polymer is evidently a promising alternative approach to the conventional physical mixture method in order to get HSEs.

**Figure 3:**
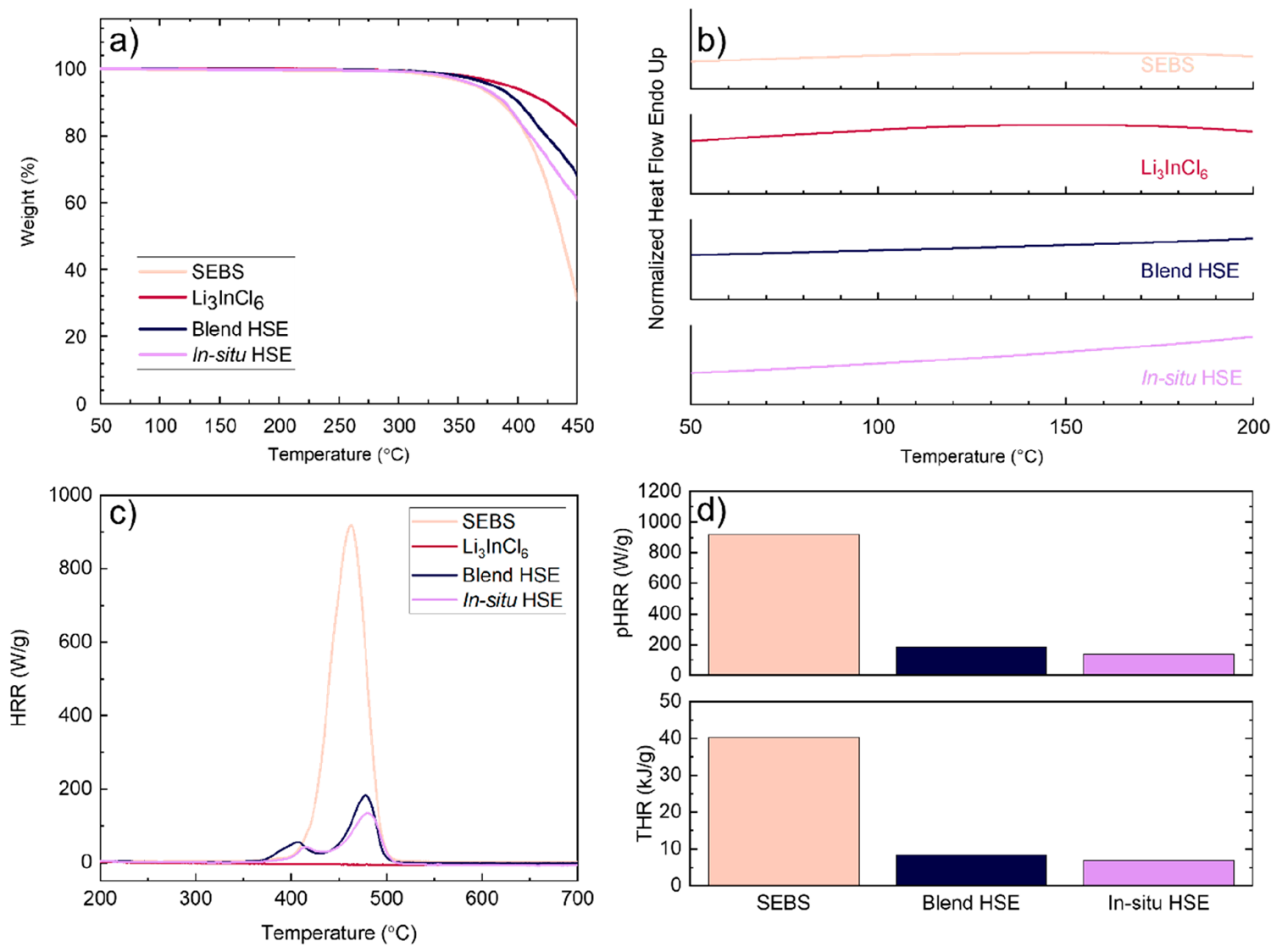
(a) TGA curves, (b) DSC thermograms, (c) HRR diagram of the microcalorimetry tests for SEBS, LIC, blend HSE, and *in situ* HSE, and (d) pHRR and THR values of SEBS, blend HSE, and *in situ* HSE.

The thermal properties of both HSEs were studied and compared to those of LIC and SEBS by TGA and DSC characterizations. Blend and *in situ* HSEs present a negligible influence on the decomposition temperature in comparison to pure LIC and SEBS. Both HSEs present excellent thermal stability up to 364 °C for *in situ* HSE and 378 °C for blend HSE, as indicated in the TGA thermograms in Figure 3a, which renders them very appealing to the *in situ* approach for automotive applications as components of ASSBs. The DSC thermograms are shown in Figure 3b. It is a commonly used technique to track the material phase transitions as a function of the temperature for polymer and inorganic materials.^32−34^ No thermal transition was detected for SEBS. Also, no phase transition was observed for LIC or any of the hybrids within the wide range of temperature, proving the successful synthesis of inorganic material and, subsequently, the stability of our hybrids. Moreover, to ensure battery safety, flame retardancy was studied by PCFS, and the results are shown in Figure 3c,d. The risk of battery fire/explosion increases when a rapid heat release is observed. Therefore, reducing the heat release rate (HRR) would make the battery safer. As shown in Figure 3c, the 80 wt % of inorganic in our hybrids increases slightly the temperature of the peak HRR (pHRR) value from 462.9 °C for the SEBS block copolymer to 477.7 °C for blend HSE and 479.9 °C for *in situ* HSE. The values of pHRR and total heat release (THR) for a pure inorganic material were negligible and hence were omitted in the materials’ comparison. The pHRR value decreases from 183.3 W/g for blend HSE to 137.9 W/g for *in situ* HSE. The presence of Li_3_InCl_6_ in the investigated hybrids significantly influences the pHRR compared to 915.8 W/g for SEBS. Additionally, the THR is also decreased from 40.2 kJ/g for the pure block copolymer to 8.3 kJ/g for blend HSE and a slightly decreased value of 6.8 kJ/g for *in situ* HSE. Therefore, *in situ* HSE shows improved flame-retardant properties compared to blend HSE prepared by conventional physical mixing.

**Figure 4:**
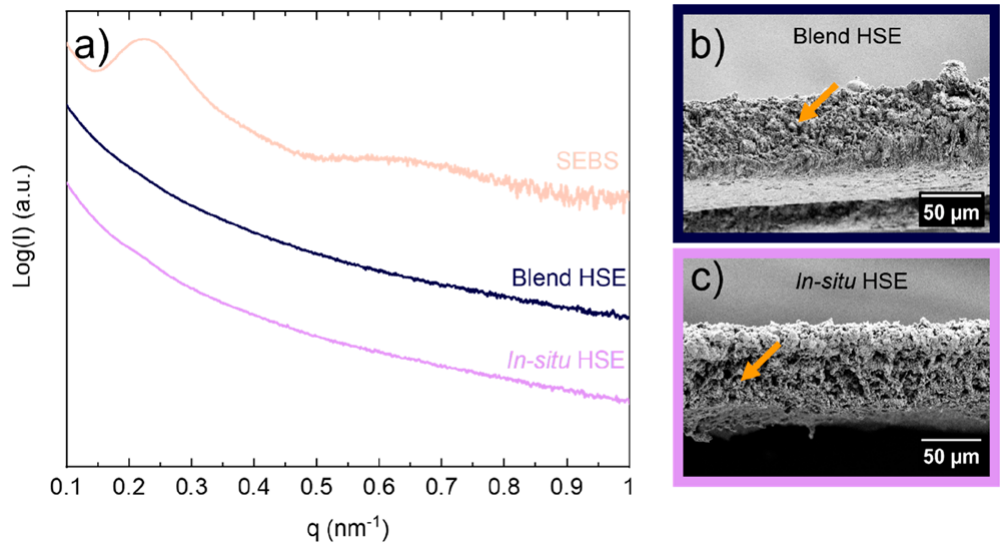
(a) SAXS profiles at RT of the SEBS block copolymer and blend and *in situ* HSEs. Cross-sectional SEM images of (b) blend and (c) *in situ* HSEs. Clusters of inorganic particles are indicated by arrows.

The morphologies of blend and *in situ* HSEs were studied by SAXS and SEM, and the results are shown in Figure 4. SAXS was performed to study the effect of the inorganic electrolyte on the morphology of the nanostructured SEBS block copolymer. We observe a primary SAXS peak at *q* = q* = 0.225 nm^–1^ for the SEBS block copolymer and a wide shoulder at *q* = 0.612 nm^–1^ at RT. Therefore, the SAXS profile of the SEBS block copolymer indicates an ordered morphology (Figure 4a).^35^ The specific assignation of a morphology is limited due to the high molecular weight of the polymer and the presence of nonuniform domain spacing in the block copolymer chain. The addition of an inorganic material (80 wt %) to the SEBS block copolymer leads to primary peak suppression in blend and *in situ* HSEs, which is consistent with the profiles expected from disordered morphologies. The inorganic electrolyte has the same effect on the morphology in both blend and *in situ* HSEs. This can be interpreted as a suppression of the effective segregation between polystyrene and polyethylene/polybutylene domains of SEBS due to the presence of Li_3_InCl_6_. The distribution of inorganic particles in the polymer matrix can be observed in the cross-sectional SEM images of our hybrids in Figure 4b,c. The presented self-standing HSEs have a thickness of 60 μm. Clusters of inorganic particles that are visible in blend HSE and *in situ* HSE present similar sizes (2–10 μm) with fewer voids between them for *in situ* HSE.

**Figure 5:**
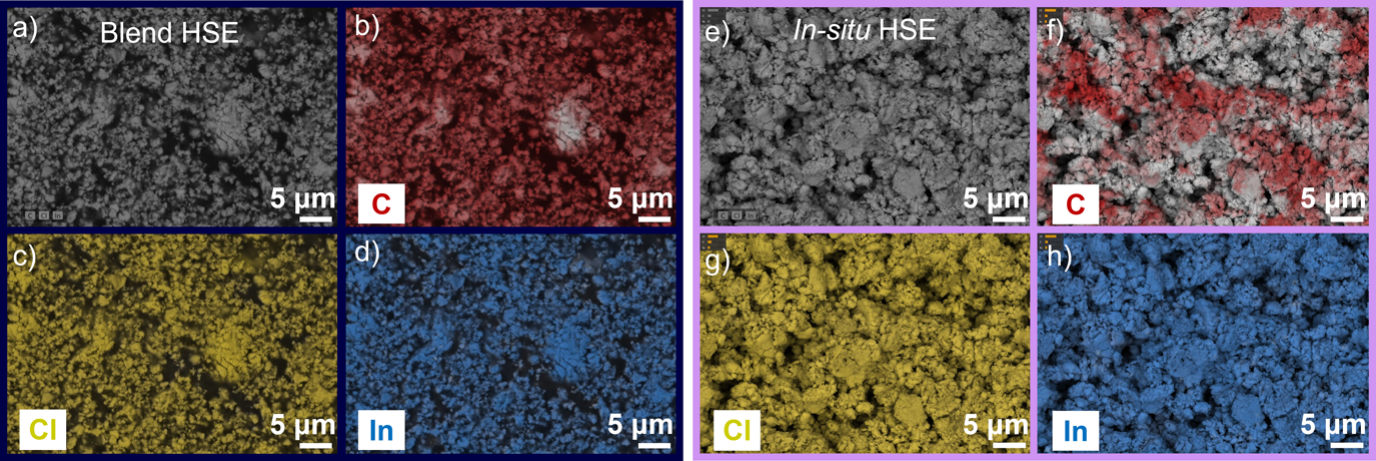
Top-view SEM images of (a) blend and (e) *in situ* HSEs and EDX mappings for (b and f) C, (c and g) Cl, and (d and h) In for blend and *in situ* HSEs, respectively. Scale bars: 5 μm.

SEM/energy-dispersive X-ray (EDX) images of blend and *in situ* HSEs were obtained to determine the homogeneity of the SEBS block copolymer in the hybrid membranes. Blend HSE (Figure 5a) shows 0.2–1-μm-sized particles that tend to form aggregates of various sizes with voids in between. The *in situ* hybrid membrane (Figure 5e) exhibits aggregates that form clusters of about 2–10 μm, which are formed by particles between 0.2 and 1 μm with relatively few voids. Further characterization is required to determine the origin of these clusters. Parts a and e of Figure 5 show the SEM images of the samples from which we performed EDX analysis. The EDX mappings of our hybrids are shown in Figure 5b–d,f–h. As expected, the spectrum is dominated by carbon (C), chlorine (Cl), and indium (In). In blend HSE, all three components are distributed uniformly, as shown in parts b (C), c (Cl), and d (In) of Figure 5. For *in situ* HSE, LIC is homogeneously distributed (Figure 5g,h); however, C from the SEBS polymer presents a random distribution, which should enable better contact between the ion-conducting inorganic phase and an electrode material (Figure 5f).

**Figure 6:**
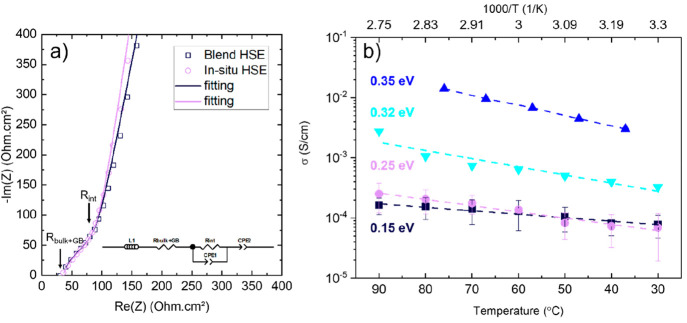
(a) Nyquist plot for HSEs at 50 °C (inset: equivalent circuit used for the fittings), (b) ionic conductivity as a function of the temperature for LIC at 360 MPa^16^ (blue ▲), LIC at 150 MPa (light-blue ▼), blend HSE at 150 MPa (■), and *in situ* HSE at 150 MPa (purple ●).

Ionic conductivity measurements were performed to study the effect of the *in situ* approach on the transport properties of HSEs. Typical EIS spectra obtained for the developed materials are shown in Figure 6a with 50 °C as an example. To calculate the ionic conductivity (σ), we used the equivalent circuit presented in the inset of Figure 6a. It assigns *R*_bulk+GB_ as the resistance of pure Li_3_InCl_6_. We assume that the high-frequency region at the intersection with the *x* axis is the sum of the bulk and grain boundary resistances of the inorganic electrolyte (*R*_bulk+GB_). The corresponding semicircles are not visible due to the frequency limit of the measurements. *R*_int_ represents the inorganic–polymer interface resistance. It is clear that *R*_int_ dominates the total resistance of the hybrids. Therefore, the interface between LIC and SEBS significantly affects the ionic conductivity, regardless of the synthetic route. The temperature dependence of the ionic conductivities of Li_3_InCl_6_ and blend and *in situ* HSEs is shown in Figure 6b. The ionic conductivities increase from 30 to 90 °C for all of the electrolytes. The ionic conductivities for pure Li_3_InCl_6_ and blend and *in situ* HSEs are 3.3 × 10^–4^, 0.8 × 10^–4^, and 0.7 × 10^–4^ S/cm at 30 °C, respectively. Blend HSE presents a lower activation energy (0.15 eV) compared to the activation energy of *in situ* HSE (0.25 eV). The difference in the energy activations might be correlated with the different grain boundary resistances between inorganic particles and, in turn, with the difference of the HSE microstructures (Figure 5).^36,37^ In general, the Li^+^ ion-conducting channels in the hybrid electrolytes with high inorganic content are through the bulk and the grain boundaries of the inorganic particles and at their interfaces when an ion-conducting polymer is used.^38,39^ The HSEs investigated in this study are based on the non-conducting SEBS polymer, eliminating the second possibility of the presence of a Li-ion transport channel.^40^ We suggest that the grain boundaries of LIC synthesized by the conventional approach are less sensitive to the temperature range compared to those synthesized by the *in situ* approach. These results indicate that the grain boundaries, and therefore, the microstructure of the hybrid electrolytes can be a key factor in controlling the energy barriers to ion transport.

**Figure 7:**
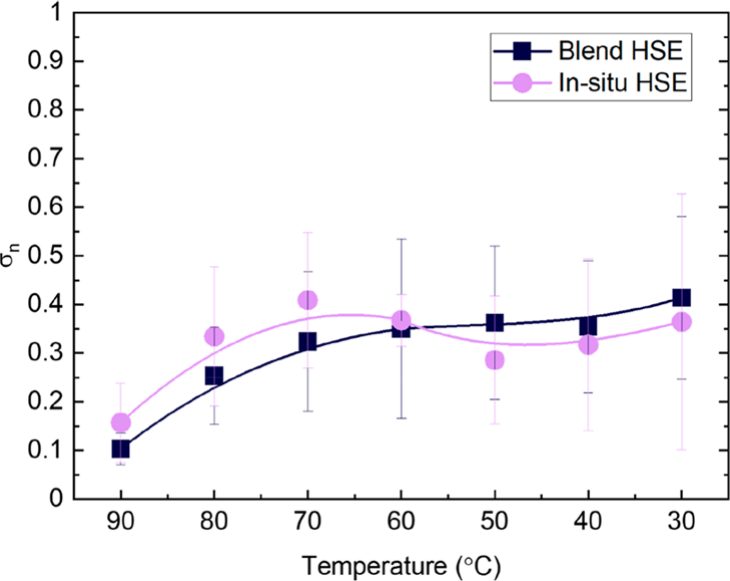
σ_n_ plotted against the temperature for HSEs.

To straighten out the complex interplay between the conductivity and structure of our hybrids, we define a normalized conductivity, σ_n_, by
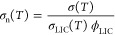
where σ_LIC_ is the ionic conductivity of pure LIC at 150 MPa and ϕ_LIC_ is the volume fraction of inorganic electrolyte. Considering the non-conducting nature of the SEBS polymer in our hybrids, we calculated σ_n_ of the hybrid electrolytes with regard to the pure conducting phase of LIC (58 vol %). In Figure 7, we plot σ_n_ versus temperature for both the blend and *in situ* hybrids. The data in Figure 7 are derived from the ionic conductivity data (Figure 6b). In Figure 7, the standard deviation was calculated by propagating the error from the hybrid conductivity data in Figure 6b. Figure 7 shows that both hybrids behave similarly, with σ_n_ decreasing as a function of the temperature. σ_n_ decreased from 0.41 ± 0.17 at 30 °C to 0.10 ± 0.03 at 90 °C for blend HSE and from 0.36 ± 0.26 at 30 °C to 0.15 ± 0.08 at 90 °C for *in situ* HSE. For ideal hybrids, σ_n_ is expected to be equal to 1. The term ideal implies that the hybrids (composed of non-conducting polymer and inorganic electrolyte) present negligible resistances between the inorganic and polymer interfaces and between inorganic grains. The conductivity of such a system is not influenced by phenomena like the grain boundary resistance, tortuosity of the system composed of inorganic and polymeric materials, etc. It is evident that σ_n_ of both hybrids presents non-ideal behavior from 30 to 80 °C, being even more unfavorable at 90 °C. This suggests that non-conducting polymers close to their *T*_g_ values have a higher impact on the resistances between the inorganic and polymer interfaces, regardless the synthetic approach. As the temperature approaches 90 °C, polystyrene and polyethylene blocks of the SEBS copolymer become softer. The σ_n_ results indicate that the interaction between the polymer and inorganic particles is increased at this temperature, decreasing σ_n_.

**Figure 8:**
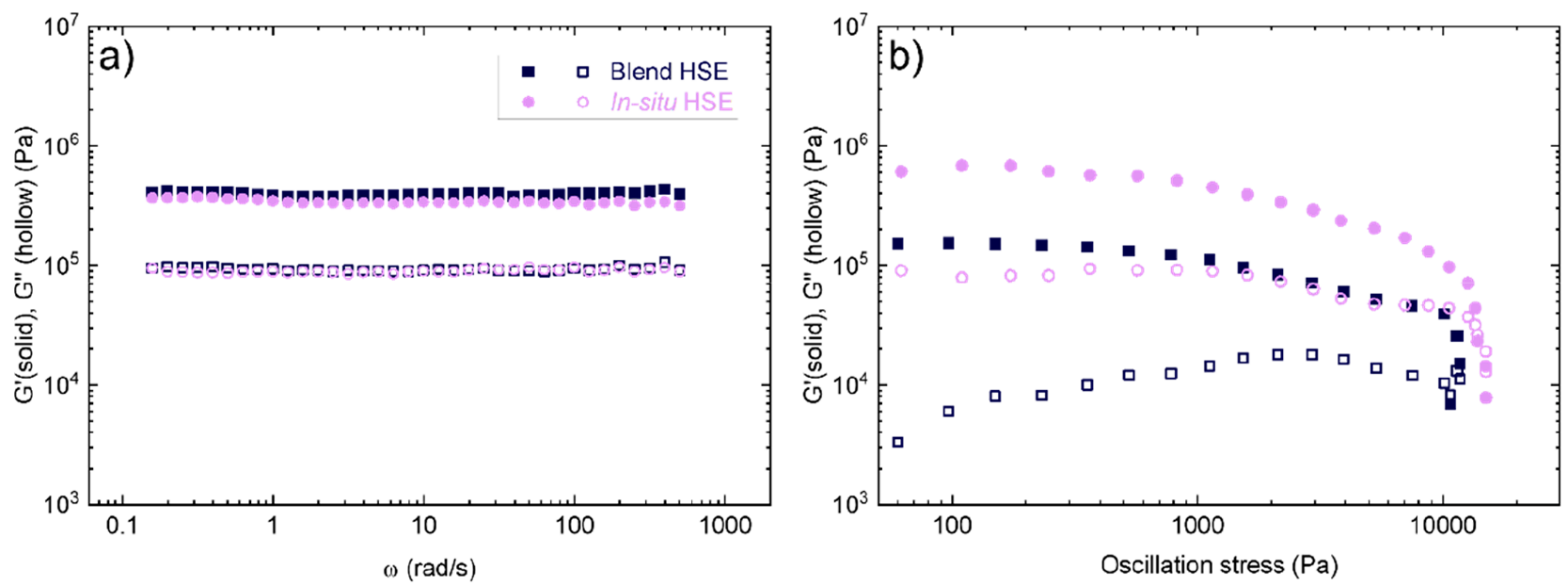
(a) *G*′ and *G*″ moduli as a function of ω at 40 °C and (b) oscillation stress dependence on *G*′ and *G*′′ at 40 °C for HSEs.

In hybrid systems, their mechanical properties are crucial to achieve an intimate contact between the electrolyte and active particles for a functioning battery. In Figure 8a, we show the frequency (ω) dependence of the storage (*G*′) and loss (*G*′′) shear moduli of the hybrid electrolytes at 40 °C. Interestingly, both of the samples present a similar range of *G*′ and *G*′′ values. The HSEs exhibit average *G*′ values of 4.02 ± 0.36 × 10^5^ and 3.43 ± 0.15 × 10^5^ Pa for blend and *in situ* HSEs, respectively. Throughout the investigated frequency window, *G*′ is much greater than *G*″, and both moduli are independent of ω. This is a distinguishing feature of elastic solids. The high inorganic content (80 wt %) of inorganic material in both HSEs allows one to achieve high values of *G*′, and the addition of SEBS (20 wt %) improves the flexibility and adhesive properties of the electrolyte, while keeping relatively high values of the ionic conductivities and making them compatible with the roll-to-roll process.

Recent studies developed by Chakraborty *et al.* have shown that the cell lifetime and failure mode are controlled by the yield stress (σ_y_).^41^ Oscillatory strain sweep experiments were performed to study the impact of the two different synthetic approaches on σ_y_. The results of these experiments are shown in Figure 8b. The point at which the storage modulus crosses the loss modulus is taken as σ_y_. The obtained values for blend and *in situ* HSEs are 11000 and 13000 Pa, respectively. These values are similar to the reported σ_y_ values for POSS–PEO–POSS/LiTFSI triblock copolymer electrolytes.

## 4 Conclusions

In summary, we have successfully demonstrated a unique approach to obtaining an *in situ* inorganic halide solid electrolyte (Li_3_InCl_6_) in the presence of a non-conducting polymer (SEBS). This practical route was compared to conventional physical mixing of the hybrid components. The investigated hybrid composition is composed of 80 wt % inorganic material and 20 wt % polymer. This innovative *in situ* approach delivers flexible self-standing membranes with high ionic conductivity (0.7 × 10^–4^ S/cm at 30 °C) and low activation energy (0.25 eV). This study also suggests that the total conductivity is dominated by the inorganic–polymer interfaces and that the microstructure of the hybrids affects the energy barriers to ion transport. The major contribution to the total resistance of the developed HSEs comes from the interface between the inorganic material and polymer binder, implying that this is the dominating factor for the total conductivity. Despite much work remaining to be done, we are confident that our research has paved the way to obtaining sustainable and cost-efficient HSEs, which can be easily implemented in the current battery manufacturing lines.

## References

[ref1] 2050 Long-Term Strategy. In-Depth Analysis in Support of the Commission Communication COM(2018) 773. A Clean Planet for All. A European Long-Term Strategic Vision for a Prosperous, Modern, Competitive and Climate Neutral Economy; European Commission, 2018 (accessed 2023–08–23).

[ref2] Mu T.; Wang Z.; Yao N.; Zhang M.; Bai M.; Wang Z.; Wang X.; Cai X.; Ma Y. Technological Penetration and Carbon-Neutral Evaluation of Rechargeable Battery Systems for Large-Scale Energy Storage. J. Energy Storage 2023, 69, 107917 10.1016/j.est.2023.107917.

[ref3] Meabe L.; Aldalur I.; Lindberg S.; Arrese-Igor M.; Armand M.; Martinez-Ibañez M.; Zhang H. Solid-State Electrolytes for Safe Rechargeable Lithium Metal Batteries: A Strategic View. Mater. Futur. 2023, 2 (3), 033501 10.1088/2752-5724/accdf3.

[ref4] Boaretto N.; Garbayo I.; Valiyaveettil-SobhanRaj S.; Quintela A.; Li C.; Casas-Cabanas M.; Aguesse F. Lithium Solid-State Batteries: State-of-the-Art and Challenges for Materials, Interfaces and Processing. J. Power Sources 2021, 502, 229919 10.1016/j.jpowsour.2021.229919.

[ref5] Goodenough J. B.; Kim Y. Challenges for Rechargeable Li Batteries. Chem. Mater. 2010, 22 (3), 587–603. 10.1021/cm901452z.

[ref6] Wang Q.; Li J.; Jin H.; Xin S.; Gao H. Prussian-Blue Materials: Revealing New Opportunities for Rechargeable Batteries. InfoMat 2022, 4 (6), 1–22. 10.1002/inf2.12311.

[ref7] Kwak H.; Wang S.; Park J.; Liu Y.; Kim K. T.; Choi Y.; Mo Y.; Jung Y. S. Emerging Halide Superionic Conductors for All-Solid-State Batteries: Design, Synthesis, and Practical Applications. ACS Energy Lett. 2022, 7 (5), 1776–1805. 10.1021/acsenergylett.2c00438.

[ref8] Wang J.; Zhao S.; Tang L.; Han F.; Zhang Y.; Xia Y.; Wang L.; Lu S. Review of the Electrochemical Performance and Interfacial Issues of High-Nickel Layered Cathodes in Inorganic All-Solid-State Batteries. Int. J. Miner. Metall. Mater. 2022, 29 (5), 1003–1018. 10.1007/s12613-022-2453-0.

[ref9] Krauskopf T.; Mogwitz B.; Hartmann H.; Singh D. K.; Zeier W. G.; Janek J. The Fast Charge Transfer Kinetics of the Lithium Metal Anode on the Garnet-Type Solid Electrolyte Li6.25Al0.25La3Zr2O12. Adv. Energy Mater. 2020, 10 (27), 2000945 10.1002/aenm.202000945.

[ref10] Zhang L.; Xu L.; Nian Y.; Wang W.; Han Y.; Luo L. Atomic Defect Mediated Li-Ion Diffusion in a Lithium Lanthanum Titanate Solid-State Electrolyte. ACS Nano 2022, 16 (4), 6898–6905. 10.1021/acsnano.2c02250. 35404580

[ref11] Li J.; Luo J.; Li X.; Fu Y.; Zhu J.; Zhuang X. Li Metal Anode Interface in Sulfide-Based All-Solid-State Li Batteries. EcoMat 2023, 5 ( (8), ) 10.1002/eom2.12383.

[ref12] Duan Y.; Bai X.; Yu T.; Rong Y.; Wu Y.; Wang X.; Yang J.; Wang J. Research Progress and Prospect in Typical Sulfide Solid-State Electrolytes. J. Energy Storage 2022, 55 (A), 105382 10.1016/j.est.2022.105382.

[ref13] Chen Y.-T.; Marple M. A. T.; Tan D. H. S.; Ham S.-Y.; Sayahpour B.; Li W.-K.; Yang H.; Lee J. B.; Hah H. J.; Wu E. A.; Doux J.-M.; Jang J.; Ridley P.; Cronk A.; Deysher G.; Chen Z.; Meng Y. S. Investigating Dry Room Compatibility of Sulfide Solid-State Electrolytes for Scalable Manufacturing. J. Mater. Chem. A 2022, 10 (13), 7155–7164. 10.1039/D1TA09846B.

[ref14] Kim K.; Park D.; Jung H. G.; Chung K. Y.; Shim J. H.; Wood B. C.; Yu S. Material Design Strategy for Halide Solid Electrolytes Li3MX6(X = Cl, Br, and I) for All-Solid-State High-Voltage Li-Ion Batteries. Chem. Mater. 2021, 33 (10), 3669–3677. 10.1021/acs.chemmater.1c00555.

[ref15] Wang C.; Liang J.; Kim J. T.; Sun X. Prospects of Halide-Based All-Solid-State Batteries: From Material Design to Practical Application. Sci. Adv. 2022, 8 ( (36), ) 10.1126/sciadv.adc9516. PMC9451152 36070390

[ref16] Li X.; Liang J.; Chen N.; Luo J.; Adair K. R.; Wang C.; Banis M. N.; Sham T.; Zhang L.; Zhao S.; Lu S.; Huang H.; Li R.; Sun X. Water-Mediated Synthesis of a Superionic Halide Solid Electrolyte. Angew. Chem. 2019, 131 (46), 16579–16584. 10.1002/ange.201909805. 31476261

[ref17] Liu H. W.; Lin C. C.; Chang P. Y.; Haw S. C.; Sheu H. S.; Chen J. M.; Chen C. C.; Jeng R. J.; Wu N. L. Reducing Oxy-Contaminations for Enhanced Li-Ion Conductivity of Halide-Based Solid Electrolyte in Water-Mediated Synthesis. J. Solid State Electrochem. 2022, 26 (9), 2089–2096. 10.1007/s10008-022-05213-y.

[ref18] Kim E.; Kim Y. Investigation of the Effect of Point Defects on the Li-Ion Conductivity of Li3InCl6. Dalt. Trans. 2022, 51 (47), 18159–18168. 10.1039/D2DT02943J. 36385654

[ref19] Chen Y.-T.; Tan D. H. S.; Ham S.-Y.; Sayahpour B.; Lee J. B.; Kim Y.; Song M.-S.; Nguyen L. H. B.; Oh J. A. S.; Ridley P.; Cronk A.; Deysher G.; Jang J.; Chen Z.; Meng Y. S. Investigating Dry Room Compatibility of Chloride Solid-State Electrolytes for Scalable Manufacturing. J. Electrochem. Soc. 2023, 170 (8), 080521 10.1149/1945-7111/acee24.

[ref20] Sedlmeier C.; Kutsch T.; Schuster R.; Hartmann L.; Bublitz R.; Tominac M.; Bohn M.; Gasteiger H. A. From Powder to Sheets: A Comparative Electrolyte Study for Slurry-Based Processed Solid Electrolyte/Binder-Sheets as Separators in All-Solid-State Batteries. J. Electrochem. Soc. 2022, 169 (7), 070508 10.1149/1945-7111/ac7e76.

[ref21] Li P.; Kim H.; Ming J.; Jung H. G.; Belharouak I.; Sun Y. K. Quasi-Compensatory Effect in Emerging Anode-Free Lithium Batteries. eScience 2021, 1 (1), 3–12. 10.1016/j.esci.2021.10.002.

[ref22] Riphaus N.; Strobl P.; Stiaszny B.; Zinkevich T.; Yavuz M.; Schnell J.; Indris S.; Gasteiger H. A.; Sedlmaier S. J. Slurry-Based Processing of Solid Electrolytes: A Comparative Binder Study. J. Electrochem. Soc. 2018, 165 (16), A3993–A3999. 10.1149/2.0961816jes.

[ref23] Tan D. H. S.; Banerjee A.; Deng Z.; Wu E. A.; Nguyen H.; Doux J. M.; Wang X.; Cheng J. H.; Ong S. P.; Meng Y. S.; Chen Z. Enabling Thin and Flexible Solid-State Composite Electrolytes by the Scalable Solution Process. ACS Appl. Energy Mater. 2019, 2 (9), 6542–6550. 10.1021/acsaem.9b01111.

[ref24] Choi H.; Kim M.; Lee H.; Jung S.; Lee Y. G.; Lee Y. M.; Cho K. Y. Unexpected Pressure Effects on Sulfide-Based Polymer-in-Ceramic Solid Electrolytes for All-Solid-State Batteries. Nano Energy 2022, 102 (July), 107679 10.1016/j.nanoen.2022.107679.

[ref25] Spencer Jolly D.; Melvin D. L. R.; Stephens I. D. R.; Brugge R. H.; Pu S. D.; Bu J.; Ning Z.; Hartley G. O.; Adamson P.; Grant P. S.; Aguadero A.; Bruce P. G. Interfaces between Ceramic and Polymer Electrolytes: A Comparison of Oxide and Sulfide Solid Electrolytes for Hybrid Solid-State Batteries. Inorganics 2022, 10 (5), 60 10.3390/inorganics10050060.

[ref26] Deiner L. J.; Gothard N. W.; Buckley J.; Clarkson D.; Greenbaum S.; Rubin I.; Noga M.; McGinn C.; Hsieh E.; Kymissis I.; Levon K. Mechanochemical Synthesis of LAGP/PEG Hybrid Solid Electrolyte: Investigation of Surface Structure and Chemistry. Solid State Ionics 2023, 394, 116191 10.1016/j.ssi.2023.116191.

[ref27] Chen S.; Wang J.; Zhang Z.; Wu L.; Yao L.; Wei Z.; Deng Y.; Xie D.; Yao X.; Xu X. In-Situ Preparation of Poly(Ethylene Oxide)/Li3PS4 Hybrid Polymer Electrolyte with Good Nanofiller Distribution for Rechargeable Solid-State Lithium Batteries. J. Power Sources 2018, 387, 72–80. 10.1016/j.jpowsour.2018.03.016.

[ref28] Huang Y. The Discovery of Cathode Materials for Lithium-ion Batteries from the View of Interdisciplinarity. Interdiscip. Mater. 2022, 1 (3), 323–329. 10.1002/idm2.12048.

[ref29] Mirmira P.; Fuschi C.; Gillett W.; Ma P.; Zheng J.; Hood Z. D.; Amanchukwu C. V. Nonconductive Polymers Enable Higher Ionic Conductivities and Suppress Reactivity in Hybrid Sulfide-Polymer Solid State Electrolytes. ACS Appl. Energy Mater. 2022, 5 (7), 8900–8912. 10.1021/acsaem.2c01388.

[ref30] Sen S.; Trevisanello E.; Niemöller E.; Shi B. X.; Simon F. J.; Richter F. H. The Role of Polymers in Lithium Solid-State Batteries with Inorganic Solid Electrolytes. J. Mater. Chem. A 2021, 9 (35), 18701–18732. 10.1039/D1TA02796D.

[ref31] Lee S. E.; Sim H. T.; Lee Y. J.; Hong S. B.; Chung K. Y.; Jung H. G.; Kim D. W. Li_6_PS_5_Cl-Based Composite Electrolyte Reinforced with High-Strength Polyester Fibers for All-Solid-State Lithium Batteries. J. Power Sources 2022, 542 (April), 231777 10.1016/j.jpowsour.2022.231777.

[ref32] Brandell D.; Mindemark J.; Hernández G. Polymer-Based Solid State Batteries; De Gruyter, 2021 10.1515/9781501521140.

[ref33] Szécsényi K. M.; Menczel J. D. DSC of Inorganic Materials. The Handbook of Differential Scanning Calorimetry; Elsevier, 2023; p 309 10.1016/B978-0-12-811347-9.00001-1.

[ref34] Li X.; Liang J.; Luo J.; Norouzi Banis M.; Wang C.; Li W.; Deng S.; Yu C.; Zhao F.; Hu Y.; Sham T. K.; Zhang L.; Zhao S.; Lu S.; Huang H.; Li R.; Adair K. R.; Sun X. Air-Stable Li3InCl6 Electrolyte with High Voltage Compatibility for All-Solid-State Batteries. Energy Environ. Sci. 2019, 12 (9), 2665–2671. 10.1039/C9EE02311A.

[ref35] Mauri M.; Floudas G.; Simonutti R. Local Order and Dynamics of Nanoconstrained Ethylene-Butylene Chain Segments in SEBS. Polymers (Basel). 2018, 10 (6), 655 10.3390/polym10060655. 30966689 PMC6404420

[ref36] Pesci F. M.; Bertei A.; Brugge R. H.; Emge S. P.; Hekselman A. K. O.; Marbella L. E.; Grey C. P.; Aguadero A. Establishing Ultralow Activation Energies for Lithium Transport in Garnet Electrolytes. ACS Appl. Mater. Interfaces 2020, 12 (29), 32806–32816. 10.1021/acsami.0c08605. 32573199

[ref37] Sakamoto J.; Rangasamy E.; Kim H.; Kim Y.; Wolfenstine J. Synthesis of Nano-Scale Fast Ion Conducting Cubic Li_7_La_3_Zr_2_O_12_. Nanotechnology 2013, 24 (42), 424005 10.1088/0957-4484/24/42/424005. 24067448

[ref38] Hu Q.; Sun Z.; Nie L.; Chen S.; Yu J.; Liu W. High-Safety Composite Solid Electrolyte Based on Inorganic Matrix for Solid-State Lithium-Metal Batteries. Mater. Today Energy 2022, 27, 101052 10.1016/j.mtener.2022.101052.

[ref39] Shi C.; Song J.; Zhang Y.; Wang X.; Jiang Z.; Sun T.; Zhao J. Revealing the Mechanisms of Lithium-Ion Transport and Conduction in Composite Solid Polymer Electrolytes. Cell Reports Phys. Sci. 2023, 4 (3), 101321 10.1016/j.xcrp.2023.101321.

[ref40] Gao H.; Grundish N. S.; Zhao Y.; Zhou A.; Goodenough J. B. Formation of Stable Interphase of Polymer-in-Salt Electrolyte in All-Solid-State Lithium Batteries. Energy Mater. Adv. 2021, 2021 10.34133/2021/1932952.

[ref41] Chakraborty S.; Sethi G. K.; Frenck L.; Ho A. S.; Villaluenga I.; Wantanabe H.; Balsara N. P. Effect of Yield Stress on Stability of Block Copolymer Electrolytes against Lithium Metal Electrodes. ACS Appl. Energy Mater. 2022, 5 (1), 852–861. 10.1021/acsaem.1c03288.

